# The *EP300:BCOR* fusion extends the genetic alteration spectrum defining the new tumoral entity of “CNS tumors with *BCOR* internal tandem duplication”

**DOI:** 10.1186/s40478-020-01064-8

**Published:** 2020-11-02

**Authors:** Arnault Tauziède-Espariat, Gaëlle Pierron, Aurore Siegfried, Delphine Guillemot, Emmanuelle Uro-Coste, Yvan Nicaise, David Castel, Isabelle Catalaa, Delphine Larrieu-Ciron, Patrick Chaynes, Amaury de Barros, Julien Nicolau, Albane Gareton, Emmanuèle Lechapt, Fabrice Chrétien, Franck Bourdeaut, François Doz, Yassine Bouchoucha, Jacques Grill, Kévin Beccaria, Nathalie Boddaert, Raphaël Saffroy, Mélanie Pagès, Pascale Varlet

**Affiliations:** 1 Department of Neuropathology, GHU Paris-Psychiatrie Et Neurosciences, Sainte-Anne Hospital, 1, Rue Cabanis, 75014 Paris, France; 2 INSERMU830, Institut Curie Research Center, Paris-Sciences-Lettres, Paris, France; 3 Laboratory of Somatic Genetics, Institut Curie Hospital, Paris, France; 4 Department of Pathology, Toulouse University Hospital, Toulouse, France; 5 INSERM U1037, Cancer Research Center of Toulouse (CRCT), Toulouse, France; 6 Université Paul Sabatier, Toulouse III, Toulouse, France; 7 UMR8203 Vectorologie Et Therapeutiques Anticancereuses CNRS, Gustave Roussy, Univ. Paris-Sud, Universite Paris-Saclay, Villejuif, France; 8 Department of Radiology, Purpan University Hospital, Toulouse, France; 9 Department of Neurology, Toulouse University Hospital, Toulouse, France; 10 Department of Medical Oncology, IUCT-Oncopole, Toulouse, France; 11 Department of Neurosurgery, Toulouse University Hospital, Toulouse, France; 12 Laboratory of Translational Research in Pediatric Oncology, SIREDO, INSERM U830, Institut Curie, Paris Sciences Lettres University, Paris, France; 13 Laboratoire de Génétique Et Biologie Des Cancers, INSERM U830, Institut Curie, Paris, France; 14 Université de Paris, Paris, France; 15 U981, Molecular Predictors and New Targets in Oncology, INSERM, Gustave Roussy, Université Paris-Saclay, Villejuif, France; 16 Département de Cancérologie de L’Enfant Et de L’Adolescent, Gustave Roussy, Université Paris-Saclay, Villejuif, France; 17 Department of Pediatric Neurosurgery, Hôpital Universitaire Necker Enfants Malades, APHP, Université de Paris, Paris, France; 18 Paediatric Radiology Department, Hôpital Necker Enfants Malades, INSERM U1163, Institut Imagine, AP-HP, University de Paris, Paris, France; 19 Department of Biochemistry and Oncogenetic, Paul Brousse Hospital, Villejuif, France

High-grade neuroepithelial tumors with the *BCOR* alteration (HGNET-*BCOR*) were isolated by a distinct methylation profile from a series of central nervous system (CNS) primitive neuroectodermal tumors (PNET) [6]. These tumors are mainly (94%, 45/48 with available molecular data) characterized by a recurrent internal tandem duplication (ITD) of the *BCOR* (*BCL6 Corepressor*) gene [1–4, 6, 9]. In rare cases, HGNET-*BCOR* presented a deletion of *BCOR* (3%, 1/48) or a mutation of the *BCOR* gene (3%, 1/48) [6]. In one case, molecular analyses failed to reveal any alteration of *BCOR* [6]*.* The cIMPACT-NOW update 6 recommends the new terminology of CNS tumor with *BCOR* ITD to designate this entity [5]. Here we report two tumors with a HGNET-*BCOR* methylation class (MC) but harboring a *BCOR* fusion with the *EP300* gene (encoding the protein p300 which is an acetyltransferase histone implicated in controlling cell growth and differentiation). The aim of our work was to compare the clinical, radiological and histopathological features of these two previously published HGNET-*BCOR* cases with ITD.

The two observations concerned a 13-year old boy (Case #1) and a 27-year-old man (Case #2). Tumors were located in the right temporal lobe (Case #1) and in the left frontal lobe (Case #2). Central neuroradiological review revealed large and well-circumscribed tumors with a meningeal attachment but without peri-lesional edema (Figs. 1 and 2). They appeared as solid hypercellular masses with a restricted apparent diffusion coefficient (ADC) in the main part of the tumors (Figs. 1 and 2). They displayed a heterogeneous enhancement after contrast injection (Figs. 1 and 2). These imaging characteristics were similar to HGNET-*BCOR* radiological data descriptions such as large and well-circumscribed tumors with a meningeal attachment, no peri-lesional edema, solid and hypercellular masses and a heterogeneous enhancement after a contrast injection [9]. Histopathological review revealed that both tumors presented the same features (Figs. 1 and 2). These tumors were mainly well-circumscribed from the brain parenchyma (with few infiltrating isolated cells at the periphery of the tumors). Pseudo-rosettes and microcysts were constantly observed. These microcysts contained a myxoid substance or occasional floating neurons. One case presented calcifications. There was intra-tumoral hetereogeneity in terms of cytology, with oligo-like, embryonal, or ependymal features. Malignancy was obvious with necrosis (calcified), high mitotic count and proliferation index, and microvascular proliferation in both cases. Immunohistochemical findings are summarized in Additional file 1: Table S1, and main features are presented in Figs. 1 and 2. There was preserved expression of H3K27me3, INI1 and ATRX in the two cases, expression of GFAP was absent, whereas Olig2 was diffusely expressed in both cases. Expression of at least one neuronal marker was present in both cases. All these results were in line with the reported HGNET-*BCOR* with ITD (25/43 reported cases were initially diagnosed as PNET) (Table 1) [1, 2, 6, 9]. Using the Heidelberg DNA methylation classifier, our two cases were classified as HGNET-*BCOR* (with calibrated max-scores of 0.6 and 0.9). RNA sequencing analysis of the two cases showed a fusion between *EP300* and *BCOR* genes, with intra exonic breakpoints (in exon 31 for *EP300*, and exon 4 for *BCOR*) (Fig. 3). None of our cases exhibited an overexpression of BCOR (Fig. 3) contrarily to 100% of reported HGNET with *BCOR* ITD [1, 2, 9]. The fusion *EP300*:*BCOR* causes the loss of the first 3 exons of *BCOR* and a part of the exon 4 encoding the Nter domain of the protein (Fig. 3). As the BCOR antibody is designed against the 300 first residues of the native protein, this epitope is missing in the resulting chimeric fusion protein and not detected by immunohistochemistry (Fig. 3).

**Fig. 1:**
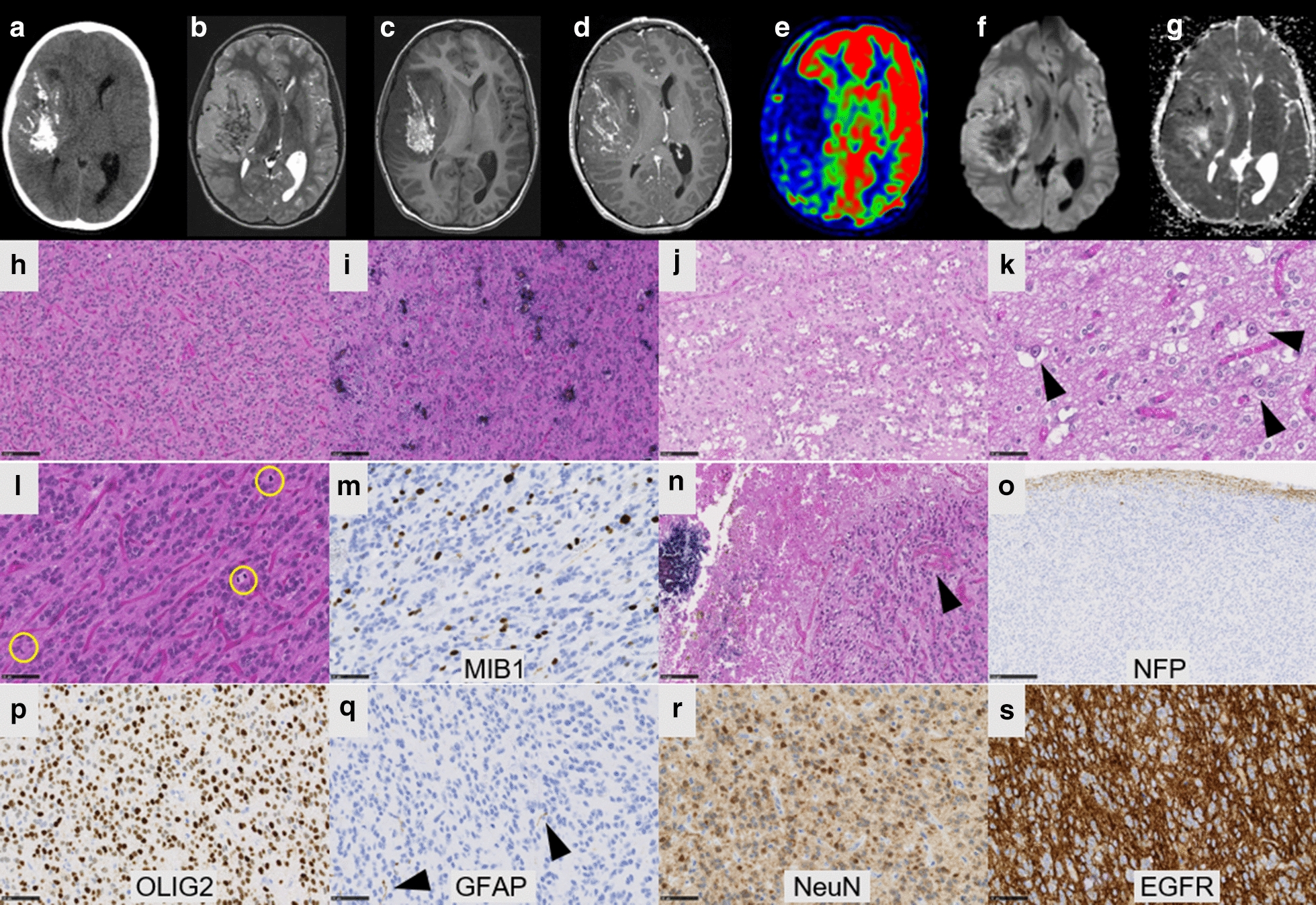
Radiological and histopathological features of #case 1. **a** Computed tomography scan showing a large and calcified tumor of the right temporal lobe. **b** T2-weighted MRI sequence reveals leptomeningeal attachment but no peri-lesional edema. **c** T1-weighted image, **d** T1-weighted image after injection of gadolinium showing a heterogeneous enhancement diffusion-weighted images. **e** Cerebral blood flow was low using arterial spin labeling. **f** Diffusion was restricted in a large part of the tumor and **g** apparent diffusion coefficient was low. **h** Compact tumor with delicate branching vessels exhibiting a chicken-wire pattern mimicking ependymoma (HPS, magnification ×200) with some calcifications (**i**, HPS, magnification ×200). **j** Microcyst formation in the tumor (HPS, magnification ×200), **k** containing occasional neuronal cells (arrowheads, HPS, magnification ×400). **l** High mitotic index (circles, HPS, magnification ×400) and **m** elevated MIB1 labeling index (magnification ×400). **n** Necrosis with calcifications, and microvascular proliferation (arrowheads, HPS, magnification ×200). **o** Well-circumscribed tumor on neurofilament staining (magnification ×100). **p** Diffuse expression of Olig2 (magnification ×400) whereas **q** GFAP was not expressed by tumor cells, with internal positive control (scattered astrocyte remnants designated by arrowheads) (magnification ×400). **r** NeuN expression by tumor cells (magnification ×400). **s** Intense EGFR expression (magnification ×400). Black scale bars represent 100 µm (**h**–**j**, **n**), and 50 μm (**k**–**m**, **p**–**s**) and 250 µm (**o**). *HPS* Hematoxylin phloxin saffron

**Fig. 2:**
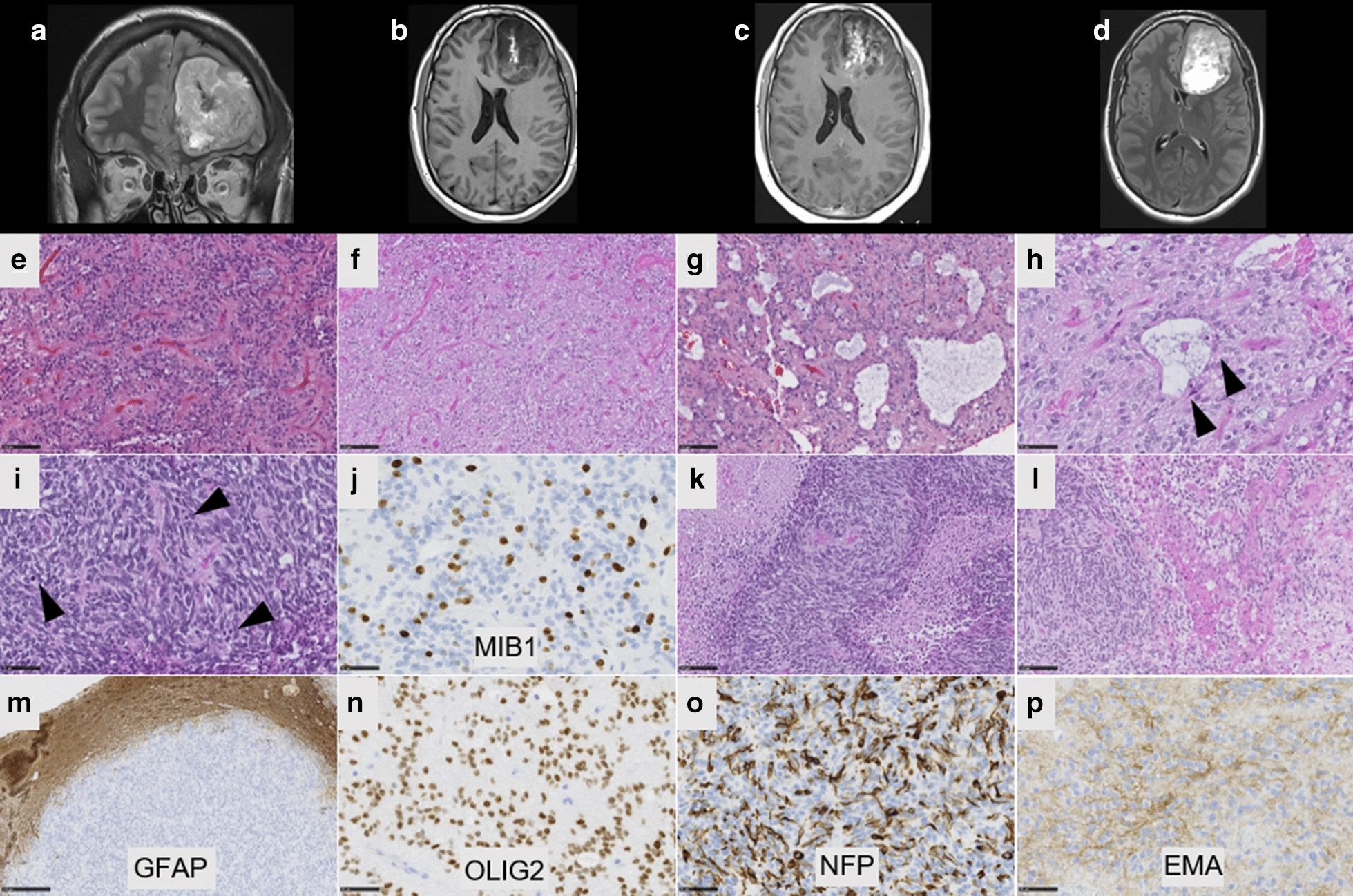
Radiological and histopathological features of #case 2. **a** Coronal T2-weighted sequence showing a large tumor without peri-lesional edema in the left frontal lobe. **b** Axial T1-weighted image showing a left frontal mass with leptomeningeal attachment and heterogeneous enhancement after gadolinium injection. **c** T1-weighted image after injection of gadolinium showing a heterogeneous enhancement. **d** Flair sequence showing hyperintensity. **e** Compact tumor with delicate branching vessels exhibiting a chicken-wire pattern (HPS, magnification ×200) with oligo-like features (**f**, HPS, magnification ×200). **g** Microcyst with a sometimes myxoid background (HPS, magnification ×200) and **h** containing some neuronal cells (arrowheads, HPS, magnification ×400). **i** Area with dense cellularity and high mitotic index (arrowheads, HPS, magnification ×400) and **j** elevated MIB1 labeling index (magnification ×400). **k** Palisading necrosis (HPS, magnification ×400) and microvascular proliferation (**l**, HPS, magnification ×400). **m** The tumor is well-circumscribed from brain parenchyma, as seen on GFAP staining, without expression in the tumor (magnification ×100). (**n**) Diffuse expression of Olig2 (magnification ×400). **o** Neurofilament expression by tumor cells (magnification ×400) and **p** cytoplasmic expression of EMA (magnification ×400). Black scale bars represent 100 µm (**e**–**g**, **k**,**l**), and 50 μm (**h**,**i**, **n**–**p**) and 250 µm (**m**)

**Table 1:** Comparison of clinical, histopathological and molecular data according to methylation classes and diagnoses

	HGNET-BCOR ITD (n = 29)	HGNET-BCOR EP300:BCOR/BCORL1 fusions (n = 3)	GLIOMAS EP300:BCOR fusion (n = 4)
Location	Infratentorial (52%)	Supratentorial (100%)	Supratentorial (100%)
Age	Median age = 3.5 YO (0;22)	Median age = 27 YO (13;72)	Median age = 12 YO (10;18)
Sex	Male (54%)	Male (100%)	Male (66%)
Radiology	Large, well-circumscribed, solid with meningeal attachment; T1 hypointense, T2 hyperintense, low ADC, heterogeneous enhancement	Large, well-circumscribed, solid with meningeal attachment; T1 hypointense, T2 hyperintense, low ADC, heterogeneous enhancement	Limited data: no meningeal attachment, not well circumscribed, T2 hyperintense, mild enhancement
Histopathology	High-grade solid tumor with perivascular pseudorosettes and microcysts	High-grade solid tumor with perivascular pseudorosettes and microcysts	Infiltrative tumor Variable grade (low in 2 cases, high in 2 cases)
Immunohistochemistry	GFAP-/Olig2+/EMA-/Neuronal markers+/BCOR+	GFAP-/Olig2+/EMA-/Neuronal markers+/BCOR-	GFAP+/Olig2+/Neuronal markers-/BCOR+
DNA-methylation class	HGNET-BCOR	HGNET-BCOR	LGG-MYB/MYBL1
Outcome	65% recurrences Median PFS = 12.5 months 30% dead at the end of follow-up Median OS = 26 months	0% recurrences 0% dead at the end of follow-up Median OS = 27 months	100% recurrences Median PFS = 4.0 months 0% dead at the end of follow-up Median OS = 7 months

*ADC* apparent diffusion coefficient, *ITD* internal tandem duplication, *OS* overall survival, *PFS* progression-free survival, *YO* years-old

**Fig. 3:**
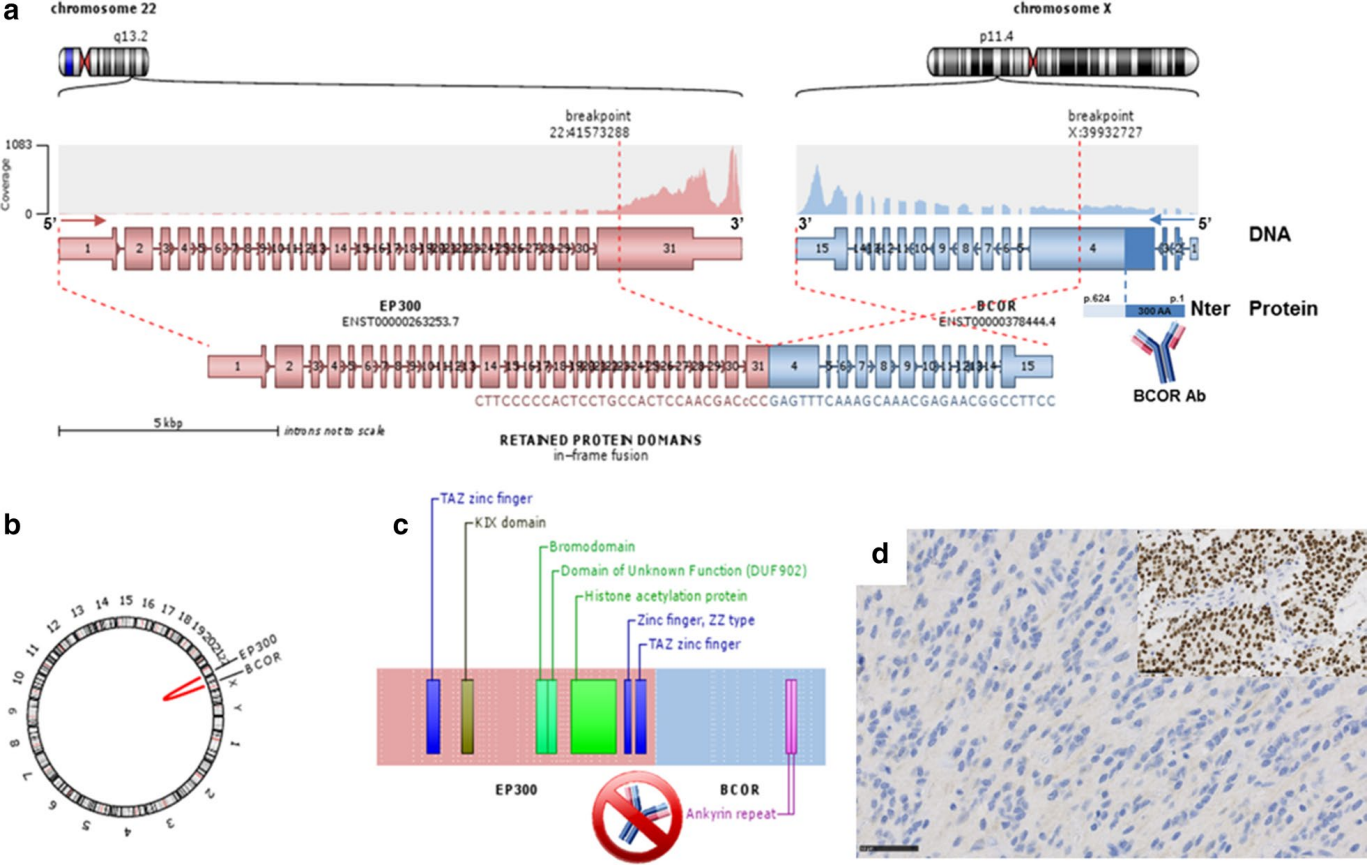
Fusion *EP300*:*BCOR* and correlation with immunohistochemistry. **a** RNAseq analysis highlights a fusion between EP300 (pink) and BCOR (blue) genes, respectively located on chr22q13.2 and chrXp11.4. As the breakpoints are intra exonic (in exon 31 for EP300, and exon 4 for BCOR), the fusion point can easily been detected by split and span reads encompassing the rearrangement with a good coverage. Localized on minus strand (inverse orientation), the DNA sequence of BCOR is switched in frame with EP300 (**b** Circos plot). This aberration causes the loss of the first 3 exons of BCOR and a part of the exon 4 encoding the Nter domain of the protein (dark blue). As the BCOR antibody is designed against the 300 first residues of the native protein and since this epitope is missing in the resulting chimeric fusion protein, it cannot be used for EP300-BCOR detection by IHC. **c** Conserved domains in the fusion protein. **d** Absence of expression of BCOR by immunohistochemistry with positive internal control (tumor of methylation class HGNET-*BCOR* with *BCOR* internal tandem duplication, insert) (magnification ×400). Black scale bars 50 μm (D)

Interestingly, this same fusion was previously reported in gliomas [7] but these cases were distinct of our cases from radiology (infiltrative pattern), histopathology and immunohistochemistry (infiltrative proliferation with calcifications, composed of GFAP positive cells without expression of neuronal markers) [7]. Moreover, gliomas described by Torre et al. were in close vicinity to LGG with an *MYB/MYBL1* alteration by t-Distributed Stochastic Neighbor Embedding plot (t-SNE) analysis whereas our cases were classified into the MC HGNET-*BCOR* and clearly clustered with HGNET-*BCOR* by t-SNE analysis (Fig. 4) [7]. Despite constant malignant histopathological features and a high rate of recurrences (65%, 17/26 cases), the prognosis of HGNET-*BCOR* with ITD remains unclear with a mortality rate of 30% (7/23 cases) [1–4, 9]. Mean/median progression-free survival (PFS) were 24.4/12.5 months and mean/median overall survival (OS) were 38.9/26.0 months in reported HGNET-*BCOR* with ITD [1–4, 9]. Notably, some reported cases were alive more than ten years after the initial diagnosis [2, 4]. In our cases, after total resection, patient #1 was treated by chemotherapy only and patient #2 was treated by chemotherapy and focal irradiation. Neither have presented a recurrence and are alive, 16 and 27 months after the initial diagnosis.

**Fig. 4:**
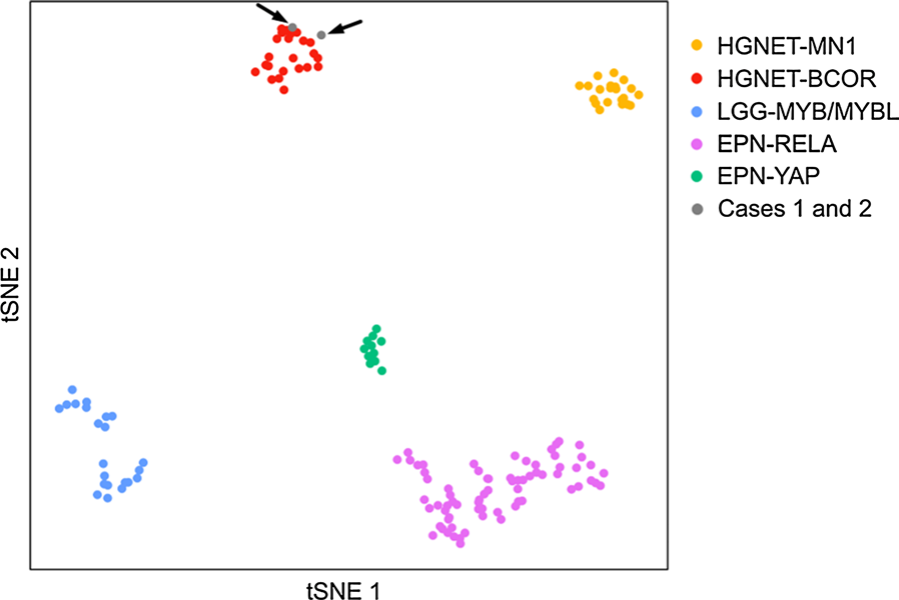
Methylation-based t-SNE distribution. The two tumors with *EP300:BCOR* fusion were compared with 147 reference samples from the Heidelberg cohort belonging to the HGNET-*BCOR,* HGNET-MN1, LGG-*MYB/MYBL,* EPN-RELA, EPN-YAP methylation classes which constitute histopathological differential diagnoses. The two cases of this study are indicated as grey dots and shown by arrows. HGNET-*BCOR*, high-grade neuroepithelial tumors with *BCOR* alteration (red dots); HGNET-MN1, high-grade neuroepithelial tumors with MN1 alteration (yellow dots); LGG-*MYB/MYBL1*, low-grade gliomas with *MYB* or *MYBL1* alteration (blue dots); EPN-RELA, ependymomas with RELA fusion (pink dots); EPN-YAP, ependymomas with YAP fusion (gree dots)

To conclude, we presented for the first time two supratentorial tumors with *EP300:BCOR* fusion sharing clinico-radiological, histopathological, immunohistochemical, and methylome homologies with HGNET-*BCOR* with ITD while they did not share similarities with the previous reported gliomas harboring this same fusion. Consequently, the *EP300:BCOR* fusion expands the spectrum of the alterations encountered in the MC HGNET-*BCOR*, and therefore, the terminology “CNS tumors with *BCOR* ITD” seems to be too restrictive. This finding echoes the data published in small round cell sarcomas of soft tissue, which may harbor *BCOR* fusions (mainly with *CCNB3* gene) and *BCOR* ITD [8]. Because the BCOR immunohistochemistry does not allow detections of HGNET-*BCOR* with fusion, we recommand searching for alternative alterations of the *BCOR* gene in the event of radiological and histopathological suspicion of this diagnosis when ITD is absent.

## Supplementary information

**Additional file 1: Table S1.** Immunohistochemical findings of our cases of HGNET-BCOR with *EP300:BCOR* fusion.
