# Supplementary material for: The EP300:BCOR fusion extends the genetic alteration spectrum defining the new tumoral entity of “CNS tumors with BCOR internal tandem duplication”
Source: Acta Neuropathol Commun. 2020 Nov 2;8:178. doi: 10.1186/s40478-020-01064-8 (PMC7607816; doi:10.1186/s40478-020-01064-8)
Supplement: Supplementary file 1 — Additional file 1: Table S1. Immunohistochemical findings of our cases of HGNET-BCOR with EP300:BCOR fusion. [file 40478_2020_1064_MOESM1_ESM.docx]

Table S1. Immunohistochemical findings of our cases of HGNET-BCOR with *EP300:BCOR* fusion

|  | **Case #1** | **Case #2** |
| --- | --- | --- |
| GFAP | - | - |
| Olig2 | +++ | +++ |
| Vimentin | +++ | +++ |
| CD56 | +++ | +++ |
| NeuN | + | + |
| Synaptophysin | - | ++ |
| Chromogranin A | - | - |
| NFP | Solid, - on tumor cells | Solid, ++ on tumor cells |
| EMA | + cytoplasmic | ++ cytoplasmic |
| CKAE1/AE3 | - | - |
| CK18 | - | - |
| S100 | ++ | - |
| SOX10 | - | - |
| Lin28A | - | - |
| Desmin | - | - |
| Myogenin | - | - |
| Smooth muscle actin | - | - |
| CD34 | - | - |
| β-catenin | Only cytoplasmic | Only cytoplasmic |
| NFκB | Only cytoplasmic | Only cytoplasmic |
| EGFR | +++ | +++ |
| p53 | + | + |
| ATRX | Preserved | Preserved |
| INI1 | Preserved | Preserved |
| BRG1 | Preserved | Preserved |
| H3K27me3 | Preserved | Preserved |

-: no expression; +: focal expression; ++: partial expression; +++: diffuse expression.
